# Lymphocyte to monocyte ratio and serum albumin changes predict tacrolimus therapy outcomes in patients with ulcerative colitis

**DOI:** 10.1038/s41598-022-17763-2

**Published:** 2022-08-09

**Authors:** Natsuki Ishida, Shinya Tani, Yusuke Asai, Takahiro Miyazu, Satoshi Tamura, Mihoko Yamade, Moriya Iwaizumi, Yasushi Hamaya, Satoshi Osawa, Takahisa Furuta, Ken Sugimoto

**Affiliations:** 1 First Department of Medicine, Hamamatsu University School of Medicine, 1-20-1 Handayama, Higashi-ku, Hamamatsu-shi, Shizuoka 431-3192 Japan; 2 Department of Laboratory Medicine, Hamamatsu University School of Medicine, Hamamatsu, Shizuoka Japan; 3 Department of Endoscopic and Photodynamic Medicine, Hamamatsu University School of Medicine, Hamamatsu, Shizuoka Japan; 4 Center for Clinical Research, Hamamatsu University School of Medicine, Hamamatsu, Shizuoka Japan

**Keywords:** Chronic inflammation, Ulcerative colitis

## Abstract

Tacrolimus therapy for ulcerative colitis is ineffective in certain patients; these patients require biologics or colectomy. We examined the ability of serum albumin levels and leukocyte subtypes to predict the therapeutic efficacy of tacrolimus. Patients with ulcerative colitis treated with tacrolimus were divided into non-failure and failure (required colectomy or switch to biologics or systemic steroids) groups. Serum albumin levels and leukocyte subtypes at induction, week 1, and week 2 after reaching high trough levels were retrospectively examined. Tacrolimus therapy failed in 18/45 patients within 3 months. The week 2/week 1 albumin ratio was significantly different between the failure and non-failure groups (P < 0.001). The receiver operating characteristic curve analysis revealed optimal cut-off value of the week 2/week 1 albumin ratio was 1.06, and area under the curve was 0.815. Analysis of leukocyte subtypes revealed significant between-group difference in the week 1 lymphocyte to monocyte ratio (P < 0.001). Multivariate analysis showed week 2/week 1 albumin ratio ≤ 1.06 and week 1 lymphocyte to monocyte ratio ≤ 3.86. Therefore**,** a low week 2/week 1 albumin and low week 1 lymphocyte to monocyte ratio predicted failure within 3 months of tacrolimus induction; a combination of these markers could accurately predict failure.

## Introduction

Ulcerative colitis (UC) is an intractable disease characterized by diarrhea, bloody stools, and abdominal pain requiring individualized treatment according to disease severity^1^. Oral tacrolimus—a calcineurin inhibitor—is an important treatment option for moderate-to-severe UC, especially for steroid-resistant or refractory patients^2,3^. Among reports of short-term and medium- to long-term outcomes of tacrolimus in UC, there are cases of tacrolimus treatment failure, and these patients must be treated with biologics or colectomy^4–7^. Currently, there are many advanced therapies for UC, including infliximab, adalimumab, and golimumab (all of which target tumor necrosis factor-α) as well as vedolizumab, tofacitinib, and ustekinumab (all of which target other molecules involved in inflammation)^8–13^. Additional treatment options for moderate-to-severe UC have made it easier for patients to switch treatments. Continuation of less effective treatment options for refractory UC leads to prolonged hospitalization and increased perioperative complications in colectomy^14^. Therefore, patients with UC who are refractory to treatment are advised to switch treatments as soon as possible; hence, an inexpensive and easily measurable predictor of treatment efficacy is needed.

Lee et al. reported that the ratio of serum albumin (Alb) levels at induction to 2 weeks after induction was a prognostic factor in anti-TNFα therapy for UC^15^. With reference to this report, we retrospectively examined the association between failure and Alb ratio at the time of tacrolimus induction and 2 weeks after induction and proved that the week 2/week 0 Alb ratio is a prognostic factor for tacrolimus treatment^16^. Additionally, Nishida et al. demonstrated that the neutrophil to lymphocyte (N/L) ratio is an independent prognostic factor after tacrolimus induction^17^. Consequently, we hypothesized that certain blood test value ratios and the ratio of change over time (before and after tacrolimus induction) may be predictive factors for tacrolimus therapy responses.

In this study, we retrospectively investigated the treatment course after induction therapy with tacrolimus and the clinical outcome-related factors. Our previous study focused only on investigating week 2/week 0 and week 1/week 0 Alb ratios^16^. In the present study, we added leukocyte subtypes, including neutrophils, lymphocytes, and monocytes, to our analysis. We also comprehensively analyzed all ratios, including the week 2/week 1 ratio, and searched for predictors of tacrolimus treatment efficacy.

## Results

### Patient characteristics

Table 1 shows the characteristics of the 45 patients with UC enrolled in this study; they did not experience failure within 2 weeks. The median age of the patients was 40 years, and the median disease duration was 1.0 year. Forty patients had extensive colitis, and five had left-sided colitis. Prior to tacrolimus induction, mucosal status was classified as Mayo endoscopic subscore (MES) 3 and MES 2 in 30 and 15 patients, respectively. The tacrolimus high trough level was reached in a median of 4 days. Eighteen (40.0%) of the enrolled patients experienced failure 3 months after tacrolimus induction. Baseline comparisons of non-failure and failure patients are shown in Table 1. There were no significant differences in characteristics, including MES and clinical activity index scores between groups.

**Table 1:** Baseline characteristics of patients.

Characteristics		All N = 45	Non-failure n = 27	Failure n = 18	P-value
Age (year), median (IQR)		40.0 (16–83)	36.0 (27.5–49.5)	60.0 (37.5–68.5)	0.062
Male/female, n (%)		35/10 (77.8/22.2)	21/6 (77.8/22.2)	14/4 (77.8/22.2)	1
Disease duration (year), median (IQR)		1.0 (0.4–4.0)	1 (0.4–2.5)	1.5 (0.3–10)	0.442
Disease extent, n (%)	Extensive colitis	40 (88.9)	25 (89.3)	16 (88.9)	1
	Left-sided colitis	5 (11.1)	3 (10.7)	2 (11.1)	
CAI (Rachmilewitz index), median (IQR)		12 (10–13)	11.3 ± 2.3	11.8 ± 3.1	0.526
MES, n (%)	MES 2	15 (33.3)	8 (29.6)	7 (38.9)	0.538
	MES 3	30 (66.7)	19 (70.4)	11 (61.1)	
Medication at study, n (%)	Oral 5-ASA	20 (44.4)	15 (55.6)	5 (27.8)	0.078
	Suppository 5-ASA	6 (13.3)	4 (14.8)	2 (11.1)	1
	Systemic steroids	28 (62.2)	18 (66.7)	10 (55.6)	0.537
	Immunomodulators	8 (17.8)	5 (18.5)	3 (16.7)	1
History of biologics, n (%)		13 (28.9)	7 (25.9)	6 (33.3)	0.739
Time to high trough level (day), median (IQR)		4 (3–8)	4 (3–8)	5.5 (3–10)	0.448

IQR, interquartile range; CAI, clinical activity index; MES, Mayo endoscopic subscore; 5-ASA, 5-aminosalicylic acid.

### Comparison of serum albumin levels and ratios between the non-failure and failure groups

We compared serum Alb levels and ratios between the non-failure and failure groups before (week 0) and after (week 1 and week 2) tacrolimus induction (see Supplementary Table S1 online, which shows serum Alb levels and ratios). A significant difference in the serum Alb level between groups occurred only at week 2 (P = 0.019). We also calculated the ratio of the three Alb levels (at week 0, week 1, and week 2) measured per patient and found that the week 2/week 0 and week 2/week 1 Alb ratios were significantly different between the groups (P = 0.013 and P < 0.001, respectively). As the week 2 value and two ratios (week 2/week 0; week 2/week 1) showed significant differences and could predict failure within 3 months, a subsequent receiver operating characteristic (ROC) analysis was performed (Table 2).

**Table 2:** Receiver operating characteristic analysis for prediction of treatment failure based on serum albumin level and ratio during the 3-month follow-up period after reaching the tacrolimus high trough level.

	Cut-off value	AUC	95% CI	Sensitivity	Specificity
Alb at week 2	3.10	0.709	0.538–0.880	72.2	70.4
Week 2/week 0 Alb ratio	1.05	0.722	0.555–0.889	61.1	81.5
Week 2/week 1 Alb ratio	1.06	0.815	0.680–0.950	77.8	81.5

AUC, area under the curve; CI, confidence interval; Alb, albumin.

**Figure 1:**
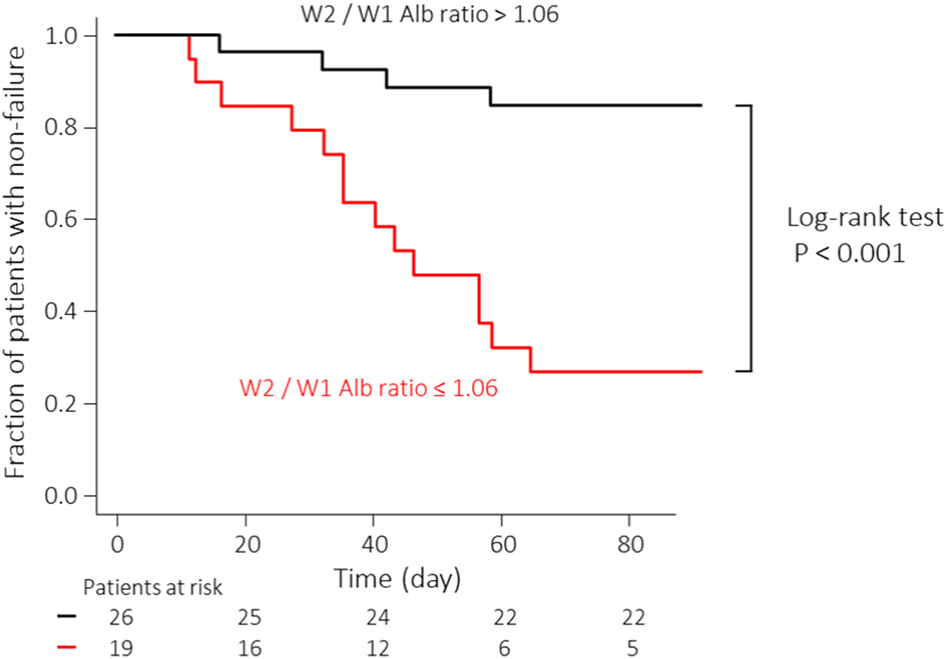
Kaplan–Meier time-to-relapse curve of patients with ulcerative colitis (UC) in relation to the week 2 (W2)/week 1 (W1) albumin (Alb) ratios > 1.06 and ≤ 1.06.

Among these three values, the week 2/week 1 Alb ratio had the largest area under the curve (AUC), and the optimal cut-off value and AUC were 1.06 and 0.815 (95% confidence interval [CI]: 0.680–0.950), respectively. Kaplan–Meier analysis was used to compare the fractions of patients with non-failure in the groups with week 2/week 1 Alb ratios > 1.06 and week 2/week 1 Alb ratios ≤ 1.06 (Fig. 1). There were 26 and 19 patients in the week 2/week 1 Alb ratios > 1.06 group and the week 2/week 1 Alb ratios ≤ 1.06 group, respectively, of whom 4 and 13 patients experienced failure during the 3-month follow-up period, respectively. The rate of failure in the week 2/week 1 Alb ratio > 1.06 group was significantly lower than that in the week 2/week 1 Alb ratio ≤ 1.06 group (log-rank test: P < 0.001).

### Comparison of leukocyte subtype absolute counts and rates between the non-failure and failure groups

**Figure 2:**
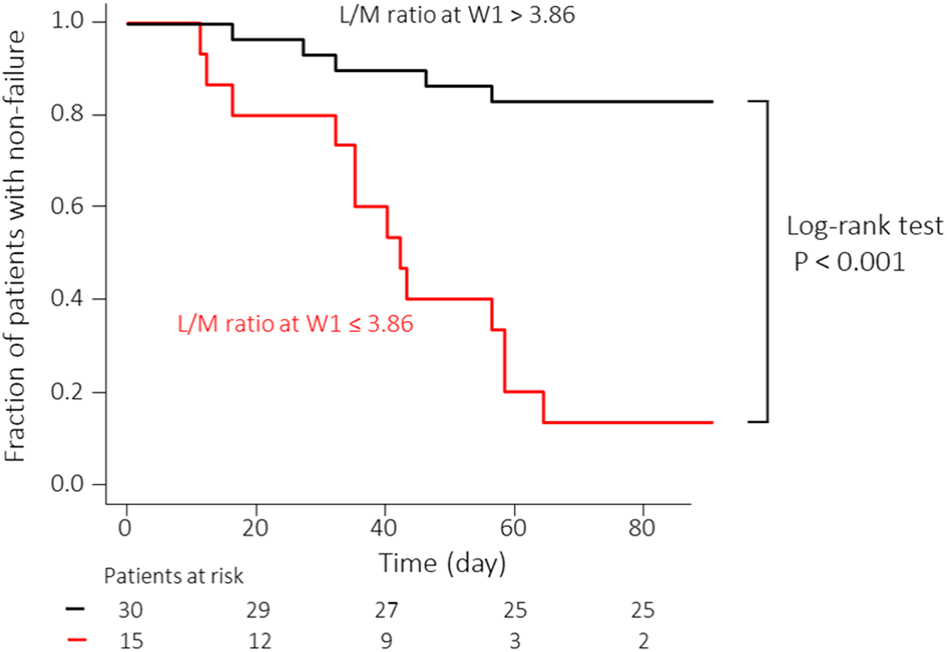
Kaplan–Meier time-to-relapse curve of patients with ulcerative colitis (UC) in relation to the leukocyte to monocyte (L/M) ratios > 3.86 and ≤ 3.86.

Similar to Alb, leukocyte subtypes were analyzed. Absolute counts and leukocyte subtype rates were compared between the non-failure and failure groups, and significant differences were found for the following values: neutrophil count at week 2 (P = 0.007), neutrophil percentage at week 2 (P = 0.002), lymphocyte count at week 1 (P = 0.013), lymphocyte count at week 2 (P = 0.003), lymphocyte percentage at week 1 (P = 0.002), and lymphocyte percentage at week 2 (P < 0.001) (see Supplementary Table S2 online, which shows the leukocyte subtype absolute counts and rates). We calculated the ratios of neutrophils, lymphocytes, and monocytes for the leukocyte subtypes and compared them between the non-failure and failure groups before and after tacrolimus induction (see Supplementary Table S3 online, which shows the leukocyte subtype ratios). The N/L ratios at weeks 1 and 2 were significantly different (P = 0.006 and P < 0.001, respectively). Although the neutrophil to monocyte ratio (N/M ratio) did not show a significant difference, the lymphocyte to monocyte ratio (L/M ratio) in week 1 and week 2 showed significant differences (P < 0.001 and P = 0.011, respectively). ROC analysis was performed on the 10 values that showed significant differences between the two groups (Table 3). The L/M ratio at week 1 showed the largest AUC, with an optimal cut-off value and AUC of 3.86 and 0.815 (95% CI: 0.674 − 0.955), respectively. The Kaplan–Meier curve was used to compare the fraction of patients without failure in the groups with an L/M ratio at week 1 > 3.86 and an L/M ratio at week 1 ≤ 3.86. During the 3-month follow-up period, four of 30 patients and 13 of 15 patients experienced failure (Fig. 2).

**Table 3:** Receiver operating characteristic analysis for prediction of treatment failure based on absolute count, rate, and subtype ratio during the 3-month follow-up period after reaching the tacrolimus high trough level.

	Cut-off value	AUC	95% CI	Sensitivity	Specificity
Neutrophil count at week 2 (/μL)	3100.5	0.737	0.579–0.894	77.8	70.4
Neutrophil rate at week 2 (%)	58.5	0.778	0.643–0.913	88.9	55.6
Lymphocyte count at week 1 (/μL)	1241.2	0.720	0.569–0.871	66.7	74.1
Lymphocyte count at week 2 (/μL)	1380.1	0.755	0.608–0.903	83.3	66.7
Lymphocyte rate at week 1 (%)	25.4	0.770	0.626–0.914	77.8	74.1
Lymphocyte rate at week 2 (%)	25.0	0.796	0.666–0.927	77.8	70.4
N/L ratio at week 1	2.89	0.744	0.596–0.892	66.7	74.1
N/L ratio at week 2	2.00	0.792	0.662–0.923	88.9	59.3
L/M ratio at week 1	3.86	0.815	0.674–0.955	72.2	92.6
L/M ratio at week 2	6.25	0.728	0.579–0.877	94.4	48.1

AUC, area under the curve; CI, confidence interval; N/L ratio, neutrophil to lymphocyte ratio; L/M ratio, lymphocyte to monocyte ratio.

### Analysis of serum Alb levels and L/M ratios as predictive factors for tacrolimus treatment outcome

**Figure 3:**
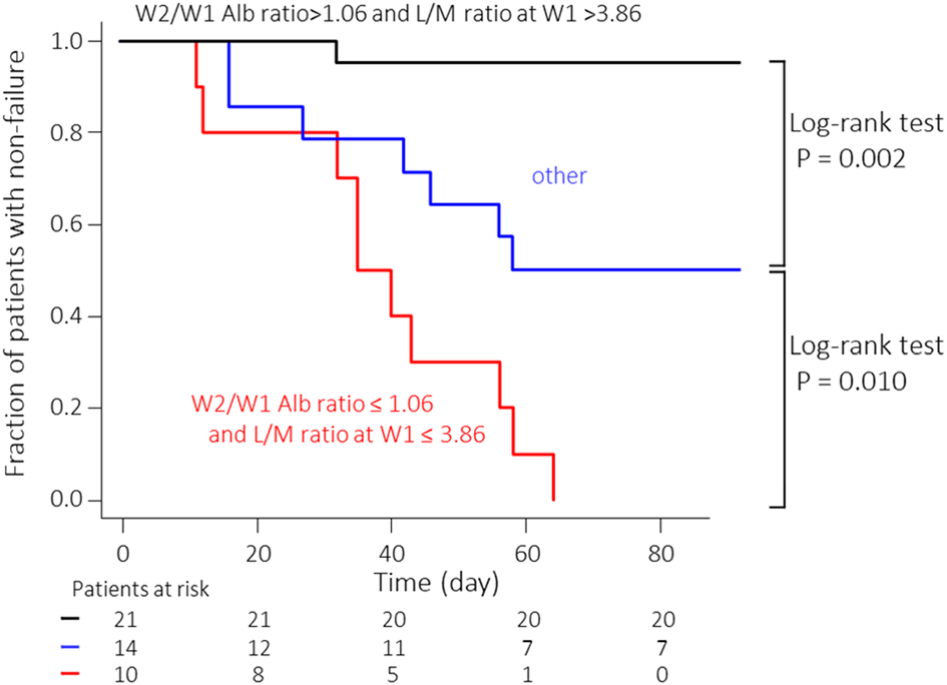
Kaplan–Meier time-to-relapse curve of patients with ulcerative colitis (UC) by group: the week 2 (W2)/week 1 (W1) albumin (Alb) ratio > 1.06 and leukocyte to monocyte (L/M) ratio > 3.86 group, the W2/W1 Alb ratio ≤ 1.06 and L/M ratio at W1 ≤ 3.86 group, and other groups (W2/W1 Alb ratio > 1.06 and L/M ratio at W1 ≤ 3.86 or W2/W1 Alb ratio ≤ 1.06 and L/M ratio at W1 > 3.86).

Kaplan–Meier analysis was performed, stratified by week 2/week 1 Alb ratio and week 1 L/M ratio cut-off values (Fig. 3). The number of patients in the week 2/week 1 Alb ratio > 1.06 and L/M ratio at week 1 > 3.86 group was 21, and one of the patients experienced failure within 3 months. In contrast, the number of patients in the other groups (week 2/week 1 Alb ratio ≤ 1.06 and L/M ratio at week 1 > 3.86 group and week 2/week 1 Alb ratio > 1.06 and L/M ratio at week 1 ≤ 3.86 group) was 14 with seven patients experiencing failure within 3 months. By contrast, all patients in the week 2/week 1 Alb ratio ≤ 1.06 and L/M ratio at week 1 ≤ 3.86 groups experienced failure within 3 months. Significant differences between the respective groups were shown using the log-rank test. Additionally, the Cox proportional hazard regression analysis was performed for week 2/week 1 Alb ratios ≤ 1.06 and week 1 L/M ratios ≤ 3.86 (Table 4). week 2/week 1 Alb ratio ≤ 1.06 and week 1 L/M ratio ≤ 3.86 were independent prognostic factors for tacrolimus treatment failure within 3 months of induction in univariate and multivariate analyses.

**Table 4:** Multivariate analysis for predicting failure 3 months following tacrolimus induction.

Variable		Univariate analysis			Multivariate analysis		
		HR	95%CI	P-value	HR	95%CI	P-value
L/M ratio at week 1 ≤ 3.43 and week 2/week 1 Alb ratio ≤ 1.05		8.325	3.172–21.85	< 0.001	6.500	1.635–25.85	0.008
Age		1.031	1.004–1.059	0.023	1.008	0.979–1.039	0.587
Sex male		1.123	0.258–3.047	0.997	0.702	0.170–2.903	0.625
Disease extent	Extensive colitis	1.123	0.258–4.888	0.877	4.046	0.412–39.72	0.230
Disease duration ≤ 1 year		1.080	1.032–1.131	< 0.001	1.060	0.989–1.137	0.100
Medication	Oral 5-ASA	0.437	0.156–1.228	0.116	0.396	0.105–1.500	0.173
	Suppository 5-ASA	0.679	0.156–2.957	0.607	1.663	0.297–9.314	0.563
	Systemic steroids	0.671	0.265–1.702	0.401	0.612	0.197–1.904	0.396
	Immunomodulators	0.840	0.243–2.903	0.783	0.566	0.095–3.391	0.533

HR, hazard ratio; CI, confidence interval; Alb, albumin; L/M ratio, lymphocyte-to-monocyte ratio; 5-ASA, 5-aminosalicylic acid.

## Discussion

We focused on serum Alb and leukocyte subtype levels as prognostic factors for tacrolimus therapy outcome in UC. Regarding treatment effect prediction using Alb levels, the change in Alb level within 2 weeks of anti-TNFα treatment can predict the prognosis^15^. We previously analyzed this idea by applying it to tacrolimus therapy and reported that the ratio of Alb at 2 weeks after achieving a high tacrolimus trough to Alb before tacrolimus induction could predict failure within 3 months^16^. In our previous study, we performed the same analysis on CRP, Hb, and WBC, excluding fractions other than Alb, but only Alb showed significant results. In our previous study, only the ratios of week 2/week 0 and week 1/week 0 Alb were calculated, not the week 2/week 1 Alb ratios. In this study, we decided to include the week 2/week 1 Alb ratio. Interestingly, the week 2/week 1 Alb ratio was a more accurate prognostic factor by ROC analysis than the week 2/week 0 Alb ratio that was reported in our previous study^16^. This difference, as described in our previous report, occurred because serum Alb levels were enriched due to intravascular dehydration associated with frequent diarrhea and bloody stools before tacrolimus induction, but serum Alb levels decreased due to blood dilution by intravenous infusion at week 1. Additionally, there are some cases that do not show sufficient Alb elevation due to a tacrolimus refractory state, and we believe that the change in Alb between week 0 and week 1 was greater than that between week 0 and week 2.

As a novel approach in this study, the absolute counts, rate, and ratio of leukocyte subtypes before tacrolimus induction and at week 1 and week 2 were analyzed by comparing the failure and non-failure groups. From these analyses, the L/M ratio at week 1 most accurately predicted failure within 3 months. The L/M ratio has been reported to be a prognostic indicator in the field of malignancy, and a low L/M ratio has been shown to be a poor prognostic factor^18^. The usefulness of the L/M ratio as an activity index in UC was first reported by Cherfane et al., and a significant difference in the L/M ratio between active and quiescent UC groups (that correlated with clinical and endoscopic activity) was shown in their study^19^. In the report by Okba et al., the L/M ratio showed a significant difference between the inactive and active UC groups^20^. Xu et al. reported that the L/M ratio in patients with UC showed a significant difference between active and inactive groups as well as between active and inactive groups in a cohort of patients with Crohn's disease^21^. As described above, the L/M ratio has been shown to be useful in assessing UC activity, and all these reports have shown that a low L/M ratio is indicative of active UC^19–21^. Previous studies of UC and Crohn's disease have shown reduced lymphocyte reactivity at the peripheral and mucosal levels, and lymphocyte reduction can occur with inflammation^22–24^. Monocytes differentiate into macrophages and dendritic cells in tissues during inflammation and play a role in innate immunity. The sustained activation of monocytes and incomplete innate immune responses can be involved in IBD development^25^. Furthermore, monocyte hyperplasia increased the risk for worse clinical outcomes in IBD^26^. The L/M ratio is a value that indicates UC activity, and it is believed to predict failure by the same mechanism as the aforementioned biomarkers. Furthermore, the reason the L/M ratio predicted failure at week 1, not at week 0 (before tacrolimus induction), in the present study was that most patients were equally active before tacrolimus induction; therefore, there were no significant differences in the L/M ratios between the failure and non-failure groups at that time. However, the L/M ratios of the non-failure group, who improved with tacrolimus treatment, increased in one week.

Nishida et al. performed a leukocyte subtype analysis similar to that conducted in our study and reported that the N/L ratio before tacrolimus induction could be a predictor of the therapeutic effect of tacrolimus^17^. In the present study, the N/L ratio before tacrolimus induction was not significantly different between the failure and non-failure groups. This difference may be because the endpoint of our study was set at 3 months while Nishida et al. did not define an endpoint. In addition, the small sample sizes in both studies may have caused the differences in data.

We further analyzed the combination of week 2/week 1 Alb and week 1 L/M ratios. Such an analysis—using a combination of two markers—has been conducted in studies on relapse prediction using multiple biomarkers; this analysis is especially seen in studies incorporating FC and FIT. These studies showed that patients with UC in clinical remission who were positive for both FC and FIT had a higher rate of subsequent relapse, demonstrating the usefulness of a combined analysis of markers^27,28^. In this study, the combination of the week 2/week 1 Alb ratio and L/M ratio at week 1 showed a more accurate failure rate.

We focused not only on the values before induction but also on the values and their changes after induction. Although it would be ideal to have a test that can predict the effect of UC treatment before induction (and there are reports of such a test), there is no test that can predict prognosis with certainty^17,29,30^. Another advantage of the evaluation method suggested in this study is that the examined Alb and leukocyte subtypes can be easily and inexpensively measured at any institution.

This study had several limitations. First, it was a single-center, retrospective study with a small sample size. Second, the examined markers have not been compared with other biomarkers. Although it was established that these biomarkers could predict the prognosis of UC, a comparison with various biomarkers will need to be conducted in our future studies. Furthermore, in this study, it took a median of 4 days from tacrolimus induction to achieve a high trough, and it took approximately 18 days from tacrolimus induction to determine both the week 2/week 1 Alb ratio and the L/M ratio at week 1; it may not be realistic to start considering the next treatment at this point. We hope that these markers will be considered as decision-making factors for tacrolimus therapy for UC^16^.

In conclusion, a low week 2/week 1 Alb ratio and a low L/M ratio at week 1 were predictive of failure of tacrolimus-based therapy for UC in this study. The combination of these markers can provide a more accurate prognosis.

## Material and methods

### Patients

Patients with UC treated with tacrolimus at our institution between August 2010 and April 2021 were enrolled in this study. The diagnosis of UC was made based on history, clinical features, and endoscopic and histological evaluation according to recent guidelines^31^. We excluded patients with inflammatory bowel disease (IBD) who were not diagnosed with UC, such as those with indeterminate colitis or unclassified IBD. Based on the clinical activity index (also called the Rachmilewitz index), biological data, and endoscopic findings, only patients with moderate-to-severe UC were included in the study. Patients who did not reach an initial high tacrolimus trough level of 10–15 ng/mL were excluded. Patients who took more than 28 days to attain the tacrolimus high trough level were excluded because biological data changes before and after induction were examined in this study.

### Study design

This was a retrospective single-center study. Tacrolimus treatment failure as indicated by switching to colectomy or biologics and corticosteroids within 3 months of tacrolimus induction was the primary outcome measure. Secondary endpoints were the predictors of tacrolimus treatment efficacy based on a comparison of biological data and clinical findings between the “non-failure” and “failure” groups.

### Disease assessment

The Rachmilewitz index was used to evaluate clinical disease activity^32^. Serum Alb and leukocyte levels were measured at our facility at admission and more than twice per week. The absolute count and rate of leukocyte subtypes, including neutrophils, lymphocytes, and monocytes, were extracted.

### Endoscopic assessment

Endoscopic examination of all patients was performed before the induction of tacrolimus. MES was used to assess the mucosal status^33^. MES was assessed using the following criteria: 0, normal or inactive disease; 1, mild disease with erythema, decreased vascular pattern, and mild friability; 2, moderate disease with marked erythema, absence of vascular patterns, friability, and erosions; and 3, severe disease with spontaneous bleeding and ulceration.

### Tacrolimus therapy and follow-up

After the initial administration, tacrolimus trough levels were measured 2–3 times per week. The dose was increased until it reached a high trough level of 10–15 ng/mL. As effective blood concentrations are reached after varying amounts of time across individuals, day 1 was defined as the time when the tacrolimus dose resulted in a high trough level for an individual. After maintaining a high trough level for 2 weeks, the dose was reduced to a low trough level (5–10 ng/mL), as previously prescribed. In this study, “failure” was defined as undergoing colectomy or switching to biologics or systemic steroids within 3 months from day 1.

### Statistical analysis

Statistical analysis was performed using SPSS version 24 (IBM, Armonk, New York, USA) and SAS version 9.4 (SAS Institute, Cary, NC). Statistical significance was set at P < 0.05. Differences between median values were compared using the Mann–Whitney U test or Fisher’s exact test. ROC analysis was conducted to determine the optimal cut-off for each value and rate for predicting failure within 3 months. The cumulative non-failure rate was analyzed using Cox proportional hazard regression and Kaplan–Meier analysis and compared using the log-rank test.

### Ethics approval

The study protocol was reviewed and approved by the Ethics Committee of Hamamatsu University School of Medicine (approval number: 20-356), and the study was performed in accordance with the 1964 Helsinki Declaration and its later amendments or comparable ethical standards. All enrolled patients provided written informed consent to participate in the study.

## Supplementary Information

Supplementary Tables.

## Data Availability

The datasets generated during and/or analysed during the current study are available from the corresponding author on reasonable request.
