# Supplementary material for: Lymphocyte to monocyte ratio and serum albumin changes predict tacrolimus therapy outcomes in patients with ulcerative colitis
Source: Sci Rep. 2022 Aug 9;12:13572. doi: 10.1038/s41598-022-17763-2 (PMC9363406; doi:10.1038/s41598-022-17763-2)
Supplement: Supplementary file 1 — Supplementary Tables. [file 41598_2022_17763_MOESM1_ESM.docx]

**Supplementary Table S1** Comparison of serum albumin levels and ratios before and after tacrolimus induction

|  | Non-failure  n = 27 | Failure  n = 18 | P-value |
| --- | --- | --- | --- |
| Alb at Week 0 (g/dL), median (IQR) | 2.90 (2.30–3.35) | 2.75 (2.00–3.50) | 0.546 |
| Alb at Week 1 (g/dL), median (IQR) | 3.00 (2.55–3.40) | 2.75 (2.12–3.38) | 0.424 |
| Alb at Week 2 (g/dL), median (IQR) | 3.50 (2.90–3.75) | 2.65 (2.15–3.25) | 0.019 |
| Week 1/Week 0 Alb ratio, median (IQR) | 1.03 (0.98–1.10) | 1.00 (0.96–1.17) | 0.898 |
| Week 2/Week 0 Alb ratio, median (IQR) | 1.14 (1.05–1.27) | 1.02 (0.95–1.13) | 0.013 |
| Week 2/Week 1 Alb ratio, median (IQR) | 1.12 (1.07–1.21) | 1.02 (0.96–1.06) | <0.001 |

Alb, albumin; IQR, interquartile range

**Supplementary Table S2** Comparison of leukocyte subtype absolute counts and rates before and after tacrolimus induction

|  | Non-failure  n = 27 | Failure  n = 18 | P-value |
| --- | --- | --- | --- |
| Neutrophil count at Week 0 (/μL), median (IQR) | 4964.9 (3969.4–7599.6) | 6445.8 (4604.4–8550.3) | 0.498 |
| Neutrophil count at Week 1 (/μL), median (IQR) | 3381.4 (2568.9–4170.5) | 4003.1 (2874.6–6981.8) | 0.218 |
| Neutrophil count at Week 2 (/μL), median (IQR) | 2533.5 (2148.1–3272.4) | 4005.30 (3137.2–5396.1) | 0.007 |
| Neutrophil rate at Week 0 (%), median (IQR) | 76.8 (63.8–82.6) | 76.5 (69.3–83.8) | 0.594 |
| Neutrophil rate at Week 1 (%), median (IQR) | 61.8 (52.8–71.8) | 70.0 (60.4–78.7) | 0.057 |
| Neutrophil rate at Week 2 (%), median (IQR) | 56.3 (47.6–66.3) | 68.5 (61.9–78.3) | 0.002 |
| Lymphocyte count at Week 0 (/μL), median (IQR) | 1016.4 (773.1–1596.0) | 1027.8 (722.3–1231.8) | 0.513 |
| Lymphocyte count at Week 1 (/μL), median (IQR) | 1761.6 (1199.4–2193.9) | 1194.8 (901.7–1590.5) | 0.013 |
| Lymphocyte count at Week 2 (/μL), median (IQR) | 1649.3 (1246.0–2304.2) | 1127.6 (937.9–1339.5) | 0.003 |
| Lymphocyte rate at Week 0 (%), median (IQR) | 12.1 (8.3–21.3) | 14.7 (7.2–18.0) | 0.594 |
| Lymphocyte rate at Week 1 (%), median (IQR) | 29.2 (23.0–42.2) | 18.5 (13.6–25.1) | 0.002 |
| Lymphocyte rate at Week 2 (%), median (IQR) | 35.0 (23.8–43.8) | 22.0 (13.9–24.8) | <0.001 |
| Monocyte count at Week 0 (/μL), median (IQR) | 378.9 (221.5–812.1) | 482.4 (308.1–817.0) | 0.900 |
| Monocyte count at Week 1 (/μL), median (IQR) | 228.5 (177.6–392.8) | 308.4 (231.6–417.1) | 0.163 |
| Monocyte count at Week 2 (/μL), median (IQR) | 300.0 (178.9–440.1) | 321.1 (263.6–520.6) | 0.254 |
| Monocyte rate at Week 0 (%), median (IQR) | 5.5 (3.6–9.0) | 6.0 (5.0–7.3) | 0.889 |
| Monocyte rate at Week 1 (%), median (IQR) | 4.3 (3.8–5.9) | 5.5 (4.0–7.0) | 0.302 |
| Monocyte rate at Week 2 (%), median (IQR) | 5.4 (4.1–7.9) | 5.9 (4.0–9.9) | 0.465 |

IQR, interquartile range

**Supplementary Table S3** Comparison of leukocyte subtype ratios before and after tacrolimus induction

|  | Non-failure  n = 27 | Failure  n = 18 | P-value |
| --- | --- | --- | --- |
| N/L ratio at Week 0, median (IQR) | 6.40 (2.47–10.43) | 4.63 (4.24–11.49) | 0.668 |
| N/L ratio at Week 1, median (IQR) | 2.12 (1.25–3.17) | 3.89 (2.39–5.77) | 0.006 |
| N/L ratio at Week 2, median (IQR) | 1.64 (1.10–2.82) | 3.04 (2.48–5.89) | <0.001 |
| N/M ratio at Week 0, median (IQR) | 12.54 (8.22–22.77) | 12.33 (9.78–16.67) | 0.972 |
| N/M ratio at Week 1, median (IQR) | 12.52 (10.07–18.74) | 12.46 (8.80–20.71) | 1 |
| N/M ratio at Week 2, median (IQR) | 9.96 (6.83–13.50) | 10.35 (6.98–16.88) | 0.711 |
| L/M ratio at Week 0, median (IQR) | 2.33 (1.33–4.64) | 2.12 (1.26–3.25) | 0.398 |
| L/M ratio at Week 1, median (IQR) | 8.38 (4.68–9.46) | 3.15 (2.47–4.71) | <0.001 |
| L/M ratio at Week 2, median (IQR) | 6.00 (3.47–9.18) | 3.74 (1.85–5.24) | 0.011 |

N/L ratio, neutrophil to lymphocyte ratio; IQR, interquartile range; N/M ratio, neutrophil to monocyte ratio; L/M ratio, lymphocyte to monocyte ratio
